# Flexible and Disposable Hafnium Nitride Extended Gates Fabricated by Low-Temperature High-Power Impulse Magnetron Sputtering

**DOI:** 10.3390/nano14141191

**Published:** 2024-07-12

**Authors:** Chia-Ming Yang, Chao-Hui Wei, Jia-Yuan Chang, Chao-Sung Lai

**Affiliations:** 1 Department of Electronic Engineering, Chang Gung University, Taoyuan City 33303, Taiwan; assa9647@hotmail.com (C.-H.W.); d0827110@cgu.edu.tw (J.-Y.C.); 2 Institute of Electro-Optical Engineering, Chang Gung University, Taoyuan City 33303, Taiwan; 3 Department of Neurosurgery, Chang Gung Memorial Hospital at Linkou, Taoyuan City 33303, Taiwan; 4 Department of Materials Engineering, Ming Chi University of Technology, New Taipei City 243303, Taiwan; 5 Department of Electronics Engineering, Ming Chi University of Technology, New Taipei City 243303, Taiwan; 6 Department of Nephrology, Chang Gung Memorial Hospital at Linkou, Taoyuan City 33303, Taiwan

**Keywords:** extended-gate field-effect transistor, flexible, HfN, HiPIMS

## Abstract

To obtain a high-performance extended gate field-effect transistor for pH detection, hafnium nitride (HfN) was first fabricated on an indium tin oxide on polyethylene terephthalate (ITO/PET) substrate using a high-power impulse magnetron sputter system (HiPIMS) in this study. It can be easily applied in biomedical diagnostic and environmental monitoring applications with the advantages of flexible, disposable, cost-effective, and reliable components. Various duty cycle conditions in HiPIMSs were designed to investigate the corresponding sensing performance and material properties including surface morphology and composition. As the duty cycle increased, the grain size of HfN increased. Additionally, X-ray photoelectron spectroscopy (XPS) analysis illustrated the presence of HfO_x_N_y_ on the deposited HfN surface. Both behaviors could result in a better pH sensing performance based on the theory of the site-binding model. Subsequently, HfN with a 15% duty cycle exhibited excellent pH sensitivity and linearity, with values of 59.3 mV/pH and 99.8%, respectively; its hysteresis width and drift coefficient were −1 mV and 0.5 mV/h, respectively. Furthermore, this pH-sensing performance remained stable even after 2000 repeated bending cycles. These results indicate the potential and feasibility of this HiPIMS-deposited HfN for future wearable chemical applications.

## 1. Introduction

Ultrahigh temperature ceramics (UHTCs) and their composite materials are usually nonmetallic inorganic materials that melt above 3000 °C or can withstand high temperatures above 2000 °C in air; these materials have excellent properties, such as excellent heat, chemical, and corrosion resistance [1,2]. These materials primarily consist of binary compounds comprising metal atoms (e.g., titanium, zirconium, and hafnium) bonded with nonmetals (e.g., carbon, nitrogen, and boron) [3]. UHTCs encompass a diverse range of nonoxide compositions, maintaining a distinct blend of metallic and ceramic properties [4]. Found in transition metal carbides, nitrides and borides, they possess strong covalent bonds and excellent properties, such as a high melting point, high hardness, excellent thermal and electrical conductivity, and excellent mechanical strength [1]. Consequently, the aerospace sector, which includes aviation, rocket propulsion, and hypersonic technology and often demands materials capable of withstanding extreme temperatures, has sparked significant interest in UHTCs [4]. Carbides and nitrides are garnering attention for their suitability in applications involving nozzles and control gases [5,6]. These materials are required to endure harsh environments characterized by high temperatures, high mechanical stresses, corrosive oxidizing conditions, and rapid heating during flight [3,4,5,7]. Fahrenholtz et al. reported the use of hafnium nitride (HfN), which has low electrical resistivity and high chemical and thermal stability and is widely used in solar applications and wear-resistant coatings [4,8]. However, the presence of structural defects such as pores, pinholes, and droplets, poses a challenge for reliability and lifetime considerations in real applications [9]. To address this issue in chemical sensor applications, this study employed high-power impulse magnetron sputtering (HiPIMS), which delivers short bursts of high-energy pulses lasting for microseconds, to fabricate a proper material quality for pH-sensing membranes. This HiPIMS technique enables a higher power density without increasing the substrate temperature [10] and a high plasma density compared to conventional sputter systems, leading to increased ionization rates of sputtered species, potentially exceeding 90% [11,12]; this results in a dense coating thin film with good crystallinity, even when processed at low temperatures [13,14].

With the increasing demand for biomedical and flexible wearable device applications, conventional glass electrodes with several drawbacks, including low flexibility and fragility, cannot be considered potential candidates [15,16,17]. Therefore, fast, robust, and simple solid-state sensing devices based on ion-sensitive field-effect transistors (ISFETs) have been proven for pH sensing in various applications, including biomedical, environmental, and industrial applications [18,19,20]. J. Van Der Spiegel proposed an extended gate field-effect transistor (EGFET) in 1983 [21], which has an extended gate connected to the gate terminal of a conventional metal–oxide field-effect transistor (MOSFET) to function as the sensing area. In comparison to conventional ISFETs, EGFETs offer many advantages, such as low cost, easy fabrication, biocompatibility, simple structure, disposability, miniaturization, facility packaging, and high sensitivity for both biomolecule and real-time detection [22]. The sensing film is a crucial component of highly sensitive and stable ISFETs and EGFETs. With recent improvements in processing technology, flexible substrates are widely used in the manufacturing of electronic devices (e.g., organic light-emitting diodes [OLEDs]) [23] and biosensors due to their suitability for point-of-care testing (POCT) diagnostics [24]. Various types of flexible substrates, including polyimide (PI)/Kapton, polyethylene terephthalate (PET), polypropylene (PP), and polyethylene naphthalate (PEN), have been extensively applied to electrochemical sensors [25]. In our previous results, ITO deposited on flexible PET substrates was applied as an EGFET with unstable pH sensing performance compared to that of other conventional EGs [26].

The purpose of this study is to provide a potential material with superior sensing performance for EGFET application by a new fabrication method, the HIPIMS. HfN films deposited through a HiPIMS with different duty cycle ratios on indium tin oxide/PET (ITO/PET) substrates working as extended gates are first presented for evaluating the sensing performance for potential applications in wearable sensors. To investigate the structural characteristics of the HfN films and their sensing performance in detail, atomic force microscopy (AFM), scanning electron microscopy (SEM), and X-ray photoelectron spectroscopy (XPS) were used to determine the surface roughness, morphology, and elemental composition, respectively. In addition, a reliability test of the sensing performance of HfN on a flexible substrate with bending cycle testing is also presented.

## 2. Materials and Methods

**Figure 1:**
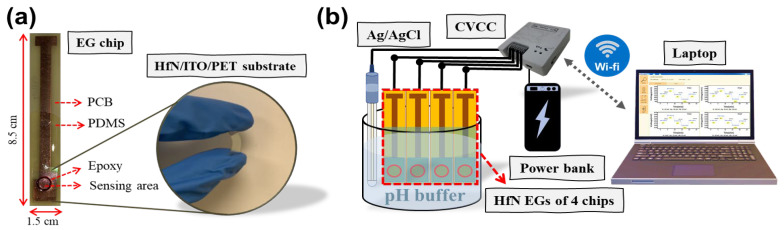
(**a**) Schematic plot of HfN EGs with a PCB package and images of a bending HfN EG, (**b**) schematic plot of HfN EGs with a CVCC readout system.

In this study, HfN extended gates (EGs) were deposited on commercial indium tin oxide and polyethylene-terephthalate (ITO/PET) substrates purchased from UNI-ONWARD, Inc., in New Taipei city, Taiwan. First, the ITO/PET substrates were cut into small pieces with dimensions of 1.5 cm × 2 cm. Before HfN deposition, areas that functioned as the bottom electrode were first covered with vacuum tape (UNI-ONWARD, 3 M-92, New Taipei city, Taiwan) on the ITO conductive thin film. Then, a HiPIMS process was performed in unipolar mode with argon (Ar) and nitrogen (N_2_), with gas flow controlled at 38 and 2 sccm, respectively, during the deposition process. A Hf target with a purity of 99.99% was obtained from Gredmann Inc. in Taipei, Taiwan. The power was set to 90 W, and the process pressure was controlled at 10 mTorr. The following experimental conditions for the HiPIMS process were selected for different duty cycles: 2.5%, 3.3%, 15%, and 25% (e.g., D2.5%, D3.3%, D15%, and D25%, respectively). The final thickness of the HfN layer in all the groups was controlled to approximately 50 nm by a time-mode control using a precalculated deposition rate. Then, an automatic dispensing system (ES2020, Ever Sharp Technology Co., New Taipei City, Taiwan) was used to define the opening area on the HfN surface with a radius of 2 mm by using chemical resistant epoxy (JC711-6, EVERWIDE, Yunlin, Taiwan). As shown in Figure 1a, for less bending and subsequent reliability concerns, HfN EG fabricated on ITO/PET was then adhered to a copper conductive line on a printed circuit board (PCB) by using silver paste (Double-O Technology, OP-928, Taipei, Taiwan). Finally, the outside of the sensing area and the copper conductive line area were all encapsulated with polydimethylsiloxane (PDMS) (SYLGARD, 184 A-B, Morrisville, PA, USA) to leave only the sensing area exposed without an electrical leakage path in the whole signal route. In addition, the deposited HfN EGs were subjected to automatic multiple bending tests and then packaged using the same method. To obtain real-time, volume, and fast measurement results, a four-channel constant voltage and constant current (CVCC) circuit readout system equipped with self-developed software was used to quickly analyze the pH sensing characteristics and long-term stability [27]. Images of the prepared HfN EGs and the connection diagram of the electrical measurement system are shown in Figure 1a,b. A semiconductor parameter analyzer (B1500A, Agilent, Santa Clarn, CA, USA) was also used to measure the typical drain-to-source current versus gate-to-source voltage (I_DS_-V_GS_) characteristics by connecting the source and drain terminal of an N-channel MOSFET (NMOS) in a commercial chip (CD4007, Texas Instruments, Pallas, TA, USA) and the gate bias through a standard Ag/AgCl reference electrode (accumet #13-620-855, Fisher Scientific, Waltham, MA, USA). The gate terminal of the NMOS was connected to the fabricated HfN EG. The settings of the operation points in the CVCC system were a drain-to-source voltage (V_DS_) fixed at 0.5 V and a drain-to-source current (I_DS_) fixed at 100 μA. Four measurement channels were connected to 4 different EGs, and one common reference electrode was connected to the ground in this system. The reference electrode and all 4 EGs were placed in the same buffer solution with a specific solution defined for experimental design purposes (e.g., sensitivity, drift, and hysteresis) to reduce variation from the environment and human errors. To verify the stability of the EGs, the drift characteristics of the sensing film surface were measured continuously for 12 h at intervals of 2.5 min in a fixed solution (e.g., a pH 7 standard buffer solution). To assess stability, hysteresis measurements were taken for the following sequence of pH buffer solutions with a pH of 7-4-7-10-7 to verify the effect of soaking in different acid and alkali solutions on the ion exchange rate of the neutral solution.

Material analysis of fabricated HfN EGs and surface treatments were performed to investigate the material properties and the potential mechanism of pH sensing improvements. First, the surface morphology of the HfN EGs was investigated via field emission scanning electron microscopy (SEM) (SU8220, Hitachi, Tokyo, Japan). The roughness of the HfN surfaces was measured using an atomic force microscope (AFM) (Nanoview 1000, FSM-Precision, Suzhou, China). The elemental distribution on the surface of the HfN EGs was analyzed by X-ray photoelectron spectroscopy (XPS) (PHI 5000 VersaProbe III, ULVAC-PHI. Inc., Kanagawa, Japan). Bending tests with automatic settings involved bending with a tensile torsion composite testing machine (YZ-11020-001, Yang Yi Technology Co., Ltd., Tainan, Taiwan). The unpackaged HfN EGs were fixed with top and bottom clamps. With an area of 1 cm × 1 cm, a moving distance of 1 mm was set to perform bending for 500, 1000, 1500, 2000, and 2500 continuous cycles.

## 3. Results

### 3.1. Surface Structure and Chemical State Analysis

**Figure 2:**
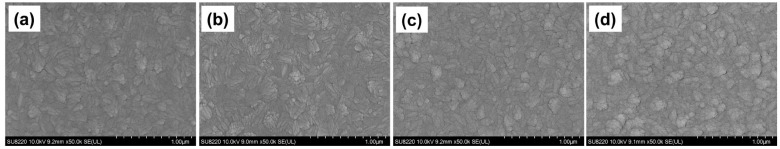
SEM images of HfN thin films deposited on ITO/PET substrates using HiPIMS with different duty cycles of (**a**) D2.5%, (**b**) D3.3%, (**c**) D15%, and (**d**) D25%.

**Figure 3:**
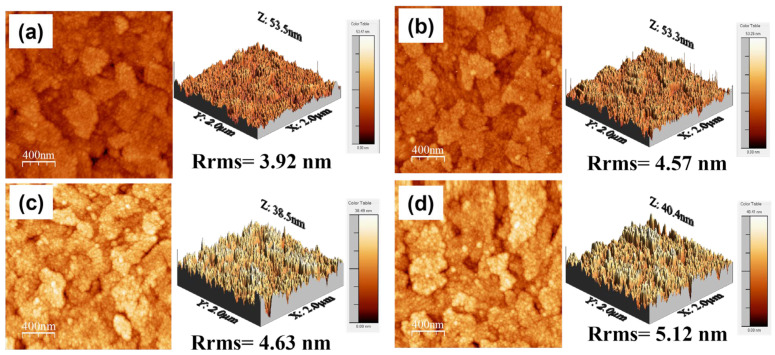
AFM results on the surface morphology of the HfN film fabricated on ITO/glass by the HiPIMS with a duty cycle of (**a**) D2.5%, (**b**) D3.3%, (**c**) D15%, and (**d**) D25% on ITO/glass substrate. The root-mean-square roughness (Rrms) is 3.92 nm, 4.57 nm, 4.63 nm, and 5.12 nm, respectively.

**Figure 4:**
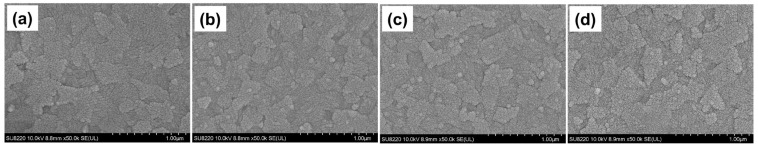
SEM images of HfN thin films deposited with ITO/glass substrates using HiPIMS with duty cycles of (**a**) D2.5%, (**b**) D3.3%, (**c**) D15%, and (**d**) D25%.

First, the surface morphology of the sensing membrane is an important factor of the sensing membrane based on the surface site number and site-binding model [28]. Due to the flexible nature of the PET substrate, the surface morphology cannot be properly investigated by atomic force microscopy (AFM). Therefore, SEM images of all four groups of HfN EGs were used to determine the surface morphologies, as shown in Figure 2a–d. The film surface is composed of grains, especially at a low duty cycle such as 2.5%; this is attributed to the high ion bombardment caused by the lower duty cycle, and more grains with high surface roughness could be deposited on the film surface [29]. Chen et al. also reported that a lower duty cycle and increased peak current or peak power density lead to an increase in the ionization rate and strong ion bombardment, resulting in a smaller grain size [30]. Liu et al. reported similar results for depositing AlCrN films with different duty cycles through a HiPIMS, with increasing roughness as the duty cycle increased [31], which supports that the minimum roughness is observed in the D2.5% group. The roughness increases with the duty cycle [31]. To verify this effect, the same HiPIMS process conditions were used on ITO/glass to determine the surface morphology by AFM. As shown in Figure 3a–d, the root-mean-square roughness (Rrms) values of the D2.5%, D5%, D15%, and D25% groups are 3.92, 4.57, 4.63, and 5.12 nm, respectively. Moreover, the SEM images show similar morphologies of HfN EGs with different duty cycles on ITO/glass, as shown in Figure 4a–d. A slight difference in the surface morphology between HfN EGs deposited on ITO/PET and those deposited on ITO/glass can be found. Liu et al. reported that the substrate surface affects the surface morphology of the film, which is attributed to the kinetic rate of atomic structure formation and affects the energy deposited during film growth [32].

**Figure 5:**
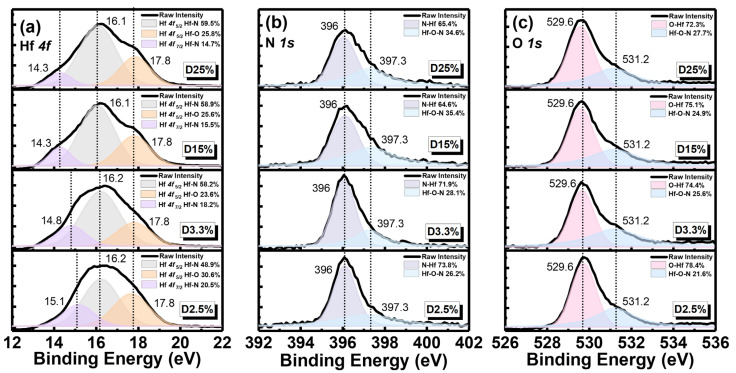
XPS spectra of (**a**) Hf 4f, (**b**) N 1s, and (**c**) O 1s binding energy diagrams for HfN EGs of D2.5%, D3.3%, D15%, and D25%, respectively.

Moreover, the composition of the sensing membrane could result in various types and numbers of surface sites, dissociation coefficients, and different sensing performances [33]. To evaluate the composition of the fabricated HfN EGs, XPS analysis was performed to study the chemical bonding of Hf 4f, N 1s, and O 1s in HfN films with different duty ratios, as shown in Figure 5a–c, respectively. As shown in Figure 5a, the XPS spectrum of the Hf 4f peak of all HfN films is composed of binding energy peaks related to Hf-N and Hf-O bonds, including Hf 4f _7/2_ and Hf 4f _5/2_. Due to spin–orbit splitting, their binding energies are approximately 14.3~15.1 eV and 16.1 eV [34,35]. Furthermore, Figure 5a shows that the binding energy peak located at 17.8 eV for the Hf-O bonds of Hf 4f _5/2_ could be evidence of the existence of HfO_2_ [34]. Zhang et al. reported similar results for Hf/HfN multilayers deposited through magnetron sputtering with an oxidized surface and with a weak HfO_2_ peak [34]. Hf atoms have a high reactivity [34,36,37] and are easily oxidized with oxygen to form HfO_x_N_y_ on the surface. Figure 5b shows the N 1s XPS spectrum of all HfN films, which can be used to confirm the existence of N 1s binding energy at 396 eV, with the N-Hf bond representing the main peak, which is consistent with the literature [34,35]. As the duty cycle increases, the ratio of the accumulated area of the Hf-O-N bond with a peak at 397.3 eV increases. This result also matches the results shown in the Hf XPS spectra. A greater number of Hf-O-N bonds could lead to better pH sensing performance because the surface site composition is sensitive to H^+^ and OH^−^ ions [33]. As shown in Figure 5c, the O 1s XPS spectrum can be seen for all HfN films. The relevant binding energy peaks include O-Hf and Hf-O-H, and their binding energies are 529.6 eV and 531.2 eV, respectively. Pan-Gam et al. reported the results of surface oxidation for DC sputtering-deposited HfN with high-temperature thermal annealing [37]. It is speculated that nitrogen in the HfN film or defects/vacancies in the crystal lattice can be easily replaced by oxygen, which has also been shown in the other literature [34,38]. Therefore, the formation of HfO_x_N_y_ compounds on the HfN surface fabricated in an Ar/N_2_ mixture by DC sputtering can be confirmed.

### 3.2. pH Sensing Characterization of HfN EGs

**Figure 6:**
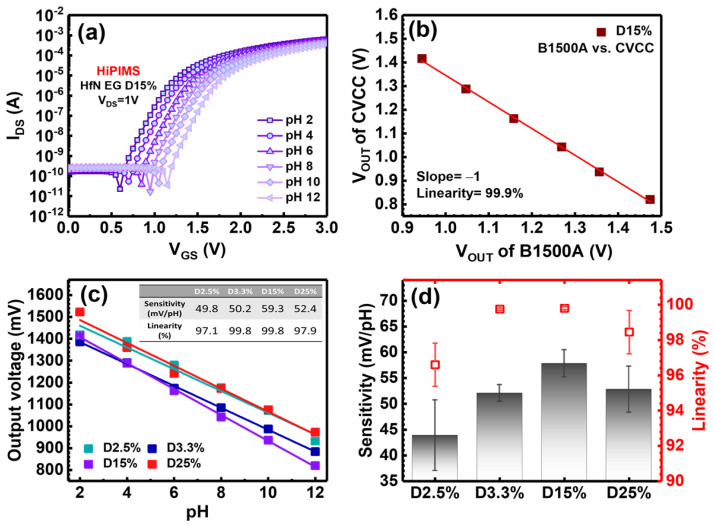
(**a**) Typical I_DS_−V_GS_ characteristics of HfN EGs of D15% with the NMOS configuration in different pH buffer solutions, (**b**) the Vout correlation between B1500A and CVCC for HfN EGs in the D15% group, (**c**) the pH sensing performance calculated by linearly fitting between the Vout from the CVCC system and the pH obtained, and (**d**) pH sensitivity and linearity statistics for all 4 groups of HfN EGs sensor.

After checking the fundamental material behavior, the pH sensing characteristics of the fabricated HfN EGs connected to NMOS with a constant V_DS_ of 0.5 V were verified by collecting I_DS_-V_GS_ curves measured in different pH buffer solutions using B1500A, as shown in Figure 6a. An evident parallel shift toward a positive voltage in the I_DS_-V_GS_ curves presented with an increasing pH, which can be attributed to the increase in the high threshold voltage (V_T_) contributed to by negative charges from surface-bound OH^−^ ions, inducing corresponding alterations in surface potential [39,40]. The V_T_ formula of the EGFET is given by Equation (1) [41]:

(1) $$V_{T}\left(EGFET\right)=V_{T}\left(MOSFET\right)-\frac{\phi }{q}+E_{ref}+\chi^{sol}-\varphi$$

where V_T_(MOSFET) is the commercial MOSFET of the threshold voltage, ϕ is the work function, q is the elementary charge, E_ref_ is the potential of the reference electrode, χ^sol^ is the surface dipole potential of the solution, and φ is the potential of the surface at the interface. With an increasing pH, the threshold voltage notably increases by φ, which was elucidated through the binding site theory and the double-layer theory [40]. The amount of charge depends on the concentration of specific ions in the solution, thereby regulating the surface charge and the formation of O^−^, OH_2_^+^, or OH groups on the surface of HfO_x_N_y_ with negative, positive, and neutral charging properties, respectively. Moreover, a conventional sensing membrane, silicon nitride (Si_3_N_4_), can have silanol (–SiOH) and amine (–SiNH_2_) sites that contribute to the potential for ion binding [42,43,44]. Similar behavior could occur in fabricated HfN EGs with a HfO_x_N_y_ surface layer. The surface charge density can be determined based on the surface morphology, material composition, and local hydrogen concentration (e.g., protonation and deprotonation of surface binding sites) [44,45]. The accumulation of OH^−^ and H^+^ ions facilitates the creation of an effective negative or positive surface potential, thereby facilitating channel formation between the source and drain at low and high gate voltages applied to the MOSFET, respectively. Based on the site-binding theory [39,40], the potential built on surface can be represented by Equation (2) [40]:

(2) $$\varphi =2.303\frac{kT}{q}\frac{\beta }{\beta +1}\left(pH_{pzc}-pH\right)$$

where q is the elementary charge, k is the Boltzmann constant, T is the absolute temperature, pH_pzc_ represents the pH at the neutral charge, and β is a sensitive film parameter defined as shown in Equation (3) [39,40]:

(3) $$\beta =\frac{2q^{2}N_{s}\sqrt{k_{a}k_{b}}}{kTC_{DL}}$$

where N_s_ is the density of the surface site, k_a_ and k_b_ are constant values for the acid and base points, respectively, and C_DL_ is the capacitance of the electrical double layer. According to site binding theory, its surface contains two different types of surface sites, namely, silanol sites and amine sites, which could both be sensitive to hydrogen ions, similar to conventional Si_3_N_4_ sensing membranes [42,46]. The site binding of HfN EGs with the surface layer of HfO_x_N_y_ can be described by Equations (4)*–*(8), which are modified from the concept of Si_3_N_4_ with a layer of SiO_x_N_y_:

(4) $$\left[{HfOH}_{2}^{+}\right]\overset{K_{+}}{↔}\left[HfOH\right]+{\left[H^{+}\right]}_{S}{;K}_{+}=\frac{\left[HfOH\right]{\left[H^{+}\right]}_{S}}{\left[{HfOH}_{2}^{+}\right]}$$

(5) $$\left[HfOH\right]\overset{K_{-}}{↔}\left[{HfO}^{-}\right]+{\left[H^{+}\right]}_{S}{;K}_{-}=\frac{\left[{HfO}^{-}\right]{\left[H^{+}\right]}_{S}}{\left[HfOH\right]}$$

(6) $$\left[Hf{NH}_{3}^{+}\right]\overset{K_{N^{+}}}{↔}\left[{HfNH}_{2}\right]+{\left[H^{+}\right]}_{S}{;K}_{N^{+}}=\frac{\left[{HfNH}_{2}\right]{\left[H^{+}\right]}_{S}}{\left[Hf{NH}_{3}^{+}\right]}$$

(7) $${\left[H^{+}\right]}_{surface}={\left[H^{+}\right]}_{bulk}exp\left(-\frac{q}{kT}\Phi \right)$$

(8) $$N_{s}=\left[{HfOH}_{2}^{+}\right]+\left[HfOH\right]+\left[{HfO}^{-}\right]+\left[Hf{NH}_{3}^{+}\right]+\left[{HfNH}_{2}\right]$$

where [H^+^]_S_ is the proton concentration at the insulator surface, [HfOH] is the amphoteric site defined as the silanol group, [HfNH_2_] is the amine group, q is the surface charge, Φ is the potential of the solid-liquid interface, and N_s_ is the density of the surface site. XPS analysis revealed that the HfN film is easily oxidized and hydrolyzed in air or in solution to form HfO_x_N_y_. Therefore, surface sites can directly interact with the electrolyte to release or combine H^+^ and OH^−^ ions for corresponding surface potential charges on the insulator surface [36,46].

To collect volume and reliable data for sensor characterization, it is crucial to have a rapid and efficient method to obtain key parameters such as sensitivity and linearity. Therefore, the output voltage (Vout) in the CVCC circuit is determined as the source-to-gate voltage based on the gate connected to ground, which is opposite to the Vout collected by the B1500A, which is defined as the gate-to-source voltage with the source connected to ground. Therefore, a good correlation with a fitting slope of −1 and a linearity of 99.9% between the Vout of the CVCC and B1500A is observed, as shown in Figure 6b. Figure 6c shows the Vout of each pH buffer solution of four different HfN EGs using a self-developed four-channel CVCC system, which is matched to the output voltage of 100 μA calculated from I_DS_-V_GS_ curves [47]. By means of the CVCC readout system, the potential for interference and human errors can be minimized by simultaneously measuring four EGs in the same pH buffer solution. As shown in Figure 6c, the Vout of each pH for the the different duty cycle groups is shown together with the linear fitting results for pH sensitivity and linearity. The sensitivities of the D2.5%, D3.3%, D15%, and D25% groups are 49.8, 50.2, 59.3, and 52.4 mV/pH, respectively. Notably, a duty cycle of 15% exhibits the highest sensitivity. Moreover, these four groups have linearities of 97.1%, 99.8%, 99.8%, and 97.9%, respectively. The lowest linearity was found for the D2.5% group, which cannot be used for pH sensing. For a clear comparison, the static data obtained from the four samples of all HiPIMS duty cycles are illustrated in Figure 6d. It is evident that the D15% group exhibits superior pH sensitivity and fitting linearity compared to the other three groups; this is speculated to be related to D15% having the largest area in Hf-O-N, as shown in Figure 5b. Table 1 lists the results of the HfN EG sensor detection of H^+^ ions compared with the sensors of other authors.

To evaluate the stability of pH sensors, a time-dependent measurement performed in the same buffer solution is commonly used, which is referred to as a drift measurement. According to the physical model of gate voltage drift proposed by Jamasb et al. [50], the surface of the sensing film will be slowly transformed to form a hydration layer over time, resulting in gradual changes in the output voltage. The change in the output voltage at different times is defined as Equation (9) [50]:

(9) $$∆V_{out}=V_{out}\left(t\right)-V_{out}(0)$$

**Figure 7:**
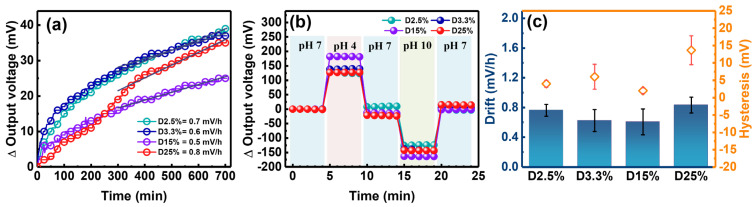
(**a**) Time−dependent behavior obtained by continuously measuring the Vout for 12 h in a pH 7 buffer solution, (**b**) the hysteresis response of the loop of pH solutions by collecting the Vout per minute five times in each pH solution step using a CVCC system, and (**c**) the statistical comparison of the calculated drift behavior and hysteresis response for HfN EGs of D2.5%, D3.3%, D15%, and D25%.

Figure 7a shows the time-dependent Vout of HfN EGs fabricated with four different duty cycles simultaneously collected in the pH 7 buffer solution using the four-channel CVCC system. The drift coefficient in the pH 7 solution is calculated by linear fitting through the relationship between the Vout and time within 5 to 12 h [51]. It is approximately 0.7, 0.6, 0.5, and 2 mV/h for the D2.5%, D3.3%, D15%, and D25% groups, respectively. This behavior suggests that all HfN EGs exhibit good stability with a small drift effect, which is comparable to that of HfO_2_ [47], RuN [48], and Ta_2_O_5_ [52] and better than that of Si_3_N_4_ [53] and SnO_2_ [54]. To test the memory effect of the previous electrolyte, hysteresis measurements can be performed in a solution cycle at a pH of 7-4-7-10-7. As shown in Figure 7b, the ΔVout of four different HfN EGs were collected every minute five times in each pH solution. By subtracting the Vout of the last point of the last pH 7 solution from that of the first, hysteresis widths of −4, 5, −1, and 14 mV were obtained for the D2.5%, D3.3%, D15%, and D25% groups, respectively. The hysteresis phenomenon can be attributed to slow-reacting surface defects and surface sites in the electrolyte solution, involving chemical interactions between OH^−^ and H^+^ions [49]. A low hysteresis width may indicate fewer defects in the sensing membrane [49], suggesting that the D15% group owns a superior pH sensing quality. This is consistent with the results shown in Figure 6c, where the D15% group demonstrates the highest pH sensitivity and the lowest hysteresis width. Figure 7c presents a statistical comparison of the drift coefficient and hysteresis width for four different duty cycle groups. It is evident that the D15% group achieves a relatively low drift coefficient and hysteresis width due to its high stability and fewer defects. Among all experimental conditions, a low hysteresis width could refer to a rapid surface response and minimal memory effect, indicative of a high-quality pH-sensing membrane. On the other hand, a high hysteresis width suggests more defects on the film surface, resulting in a delayed response and memory effect, thereby the practical applications of the sensor [49].

### 3.3. Reliability Analysis of the Bending Cycles

**Figure 8:**
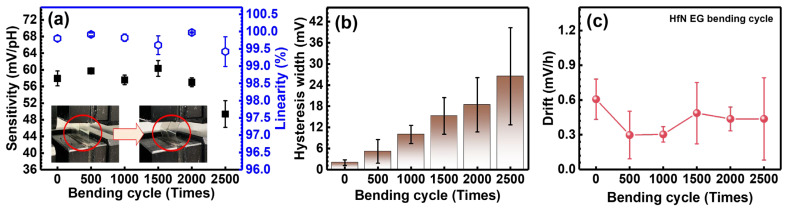
Sensing performance of HfN EGs of D15% treated with bending cycle tests: (**a**) sensitivity and linearity measured in buffer solutions ranging from a pH of 2 to 12, (**b**) hysteresis width of the loops in a pH buffer solution, and (**c**) drift behavior in a pH 7 buffer solution for 12 h.

**Figure 9:**
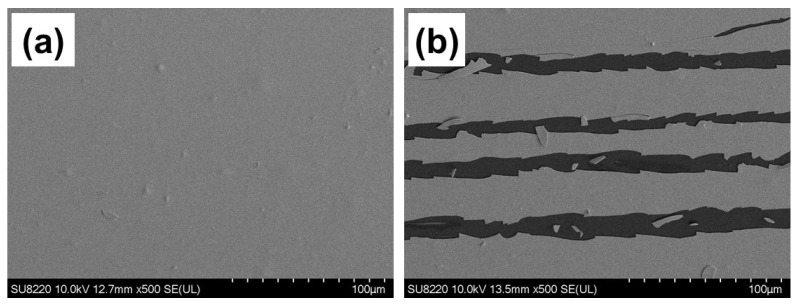
SEM images of HfN EGs with D15% (**a**) before and (**b**) after 2500 bending cycles.

To evaluate the bendability of the flexible pH sensor, the fabricated flexible HfN EG sensor was subjected to automatic mechanical bending cycle tests, as shown in the inset of Figure 8a. As shown in Figure 8a, the pH sensitivity was determined after bending the HfN EGs of the D15% group with tensile torsion for 500, 1000, 1500, 2000, and 2500 cycles. The sensitivity decreased significantly to only 49.3 mV/pH, and the linearity decreased slightly to 99.4% at 2500 cycles. Figure 8b shows that the hysteresis width begins to increase after 2000 bending cycles. Figure 8c shows that the drift effect after bending is comparable to or slightly lower than that before bending. It is speculated that pores are created on the surface of the film after bending, and ions run into the pores on the surface of the film, compensating for the original drift caused by the hydration effect. To verify the impact of the decreased sensitivity caused by bending, the surface morphology of the bending area was analyzed using SEM. As shown in Figure 9a, the surface of the HfN EGs is flat. Many cracks were found on the surface that was bent 2500 times, as shown in Figure 9b, which can be used to properly explain the changes in sensing performance. Currently, the maximum number of bending cycles is suggested to be less than 2000, which is suitable for disposable sensors.

## 4. Conclusions

To have a high-performance extended gate field-effect transistor for pH detection, this study presents an investigation of the sensing characteristics of HfN thin films deposited by adjusting the duty cycle through a HiPIMS from 2.5% to 25% throughout a whole low-temperature process. The electrical measurement results indicate that the superior pH sensitivity and linearity of HfN EGs with D15% are 59.3 mV/pH and 99.8%, respectively. The drift behavior and hysteresis response were 0.5 mV/h and -1 mV, respectively, which are stable for real applications. The pH sensing performance of HfN EGs could be explained by the high density of Hf-O-N binding sites on the HfO_x_N_y_ surface, as illustrated by the XPS spectra. Furthermore, HfN EGs with D15% retained stable sensing behavior within 2000 cycles in the bending test. The SEM image shows that cracks appeared on the surface of the specimen after bending for 2500 cycles, leading to a decrease in the HfN EG sensing performance. This fabricated HfN EG on ITO/PET at low temperatures has acceptable pH sensing performance, and bending durability could be suggested for wearable or disposable devices to detect various biomarkers for use in medical diagnostics.

## Figures and Tables

**Table 1:** Comparison of sensor data for various materials in pH buffer solutions, including sensitivity and linearity.

Sensing Film	Device	Substrate	Method	Background Solution	Sensitivity (mV/pH)	Linearity (%)	Ref.
IrO_x_	EGFET	Polyimide	Sol-gel	pH 1.5–12	51.1	95.3	[15]
Si_3_N_4_	ISFET	Undoped silicate glass (USG)	Sputter	pH 4–7–9	46	N/A	[18]
ITO	EGFET	PET	Sputter	pH 2–12	50.1	99	[26]
RuN	EGFET	Si	Sputter	pH 1–13	58.03	N/A	[48]
CuS	EGFET	Glass	Spray pyrolysis deposition	pH 2–12	24	98.18	[49]
HfN	EGFET	ITO/PET	HiPIMS	pH 2–12	59.3	99.8	This work

## Data Availability

The data presented in this study are available on request from the corresponding authors.
